# *Stelis zootrophionoides* (Orchidaceae: Pleurothallidinae), a New Species from Mexico

**DOI:** 10.1371/journal.pone.0048822

**Published:** 2012-11-07

**Authors:** Sergio E. Ramos-Castro, Miguel Castañeda-Zárate, Rodolfo Solano-Gómez, Gerardo A. Salazar

**Affiliations:** 1 Departamento de Ecología Evolutiva, Instituto de Ecología, Universidad Nacional Autónoma de México, Mexico, DF, Mexico; 2 Departamento de Botánica, Instituto de Biología, Universidad Nacional Autónoma de México, Mexico, DF, Mexico; 3 Instituto Politécnico Nacional, Centro Interdisciplinario de Investigación para el Desarrollo Integral Regional unidad Oaxaca, Oaxaca, Mexico; 4 Asociación para la Conservación de Orquídeas Silvestres, Facultad de Biología, Xalapa, Veracruz, Mexico; Centro de Investigación y de Estudios Avanzados, Mexico

## Abstract

**Background:**

*Stelis* (Orchidaceae) encompasses approximately 1100 species of epiphytic orchids distributed throughout the Neotropics, with the highest diversity in Andean South America. Sixty-two species were recorded previously in Mexico.

**Methods:**

We formally describe here *Stelis zootrophionoides* as a new species from Chiapas, Mexico. To determine its systematic position, we conducted a morphological comparison with other members of Pleurothallidinae and a phylogenetic analysis of nucleotide sequences from the plastid *matK/trnK* and *trnL/trnF* regions, as well as the nuclear ribosomal ITS region for 52 species of Pleurothallidinae. Sequences of 49 species were downloaded from GenBank and those of three species, including the new taxon, were newly generated for this work. The new species is described and illustrated; notes on its ecological preferences and a comparison with closely related species are presented.

**Conclusions:**

The new species, known only from one location and apparently restricted to the cloud forest in the central highlands of Chiapas, Mexico, is considered a rare species. This small epiphyte is unique among the Mexican species of *Stelis* by the combination of dark purple flowers with the distal third of the dorsal sepal adhered to the apices of the lateral sepals, which are partially united into a bifid synsepal, leaving two lateral window-like openings, and sagittate labellum. *Stelis jalapensis*, known from southern Mexico and Guatemala, also has the apices of the sepals adhered to each other, but it is distinguished by its larger flowers with lanceolate, acute dorsal sepal, completely fused lateral sepals (i.e. the synsepal is not bifid), and oblong-elliptic labellum. The phylogenetic analysis shows that *S. zootrophionoides* is closely related to other Mexican *Stelis* and corroborates previous suggestions that fused sepal apices have arisen independently in different lineages of Pleurothallidinae.

## Introduction

*Stelis* Sw. (Orchidadeae) is the most species-rich genus in subtribe Pleurothallidinae [1], [2]. However, the number of species recognized varies depending on the classification. According to Luer [1], [2], the genus includes ∼900 species, whereas in the circumscription of Pridgeon and Chase [3] there are almost 1100 species.

*Stelis* species are epiphytic, lithophytic, or rarely terrestrial orchids restricted to the American tropics, distributed from southwest Florida and northwestern México through the Antilles to Bolivia and Brazil, with the highest diversity concentrated in Andean South America [1], [2]. The taxa occur in a wide variety of habitats, from sea level to 4000 m in elevation [3]. Sixty-two species of *Stelis* were recorded previously from Mexico [4], [5], [6]. Recently, during a field trip in the central highlands of Chiapas, we collected specimens of a small plant belonging to subtribe Pleurothallidinae in which the distal part of the dorsal sepal was adhered to the apices of the lateral sepals, but otherwise was separated from them to form two lateral window-like openings. Species with similar floral features are known to occur in several unrelated genera of Pleurothallidinae, such as *Acianthera* Scheidw., *Phloeophila* Hoehne & Schltr., *Specklinia* Lindl., *Stelis* Sw., and *Zootrophion* Luer. After a comparison of the newly collected plant with all the species of Pleurothallidinae known from México and Central America, it was evident that it represents an unknown species, which is described and illustrated here for the first time.

## Materials and Methods

### Ethics statement

We collected material in the Reserva Ecológica Cerro Huitepec Chiapas, Mexico, which is a protected area that is privately owned by Pronatura Mexico, AC. Pronatura permits research in their reserves and no specific permits are required for field studies like the one described here.

### Morphological observations

The description of the new species was based on examination under a stereomicroscope of fresh, pickled, and pressed specimens of the new species. A line drawing was prepared from fresh material with the aid of a drawing tube adapted to the stereomicroscope. The morphological comparison with other species of Pleurothallidinae was based on study of live plants in the field and in cultivation, herbarium specimens, and information gathered from the literature.

### Sampling

We downloaded from GenBank sequences of 49 species belonging to 30 genera of subtribe Pleurothallidinae sensu Pridgeon et al. [3]. Additionally, for this work we sequenced three species, *Stelis nigriflora* (L.O.Williams) Pridgeon & M.W.Chase, *S. rubens* Schltr., and the new species. We used *Dilomilis montana* Summerh. as outgroup, following previous phylogenetic studies [6]. GenBank accessions for all the DNA sequences and voucher information for the new ones are given in Table S1. The aligned matrix is available on request from G.A.S. (gasc@ibunam2.ibiologia.unam.mx).

### Molecular markers

We analyzed the nucleotide sequences of the plastid *matK/trnK* and *trnL/trnF* regions as well as the nuclear ribosomal ITS region. All these regions have been used previously to infer phylogenetic relationships among the Pleurothallidinae [7] and other orchid lineages [8]. Genomic DNA was extracted from fresh plant tissue using a 2× cetyltrimethylammonium bromide (CTAB) protocol based on Doyle and Doyle [9] modified by the addition of 2% polyvinyl pyrrolidone (PVP) to the extraction buffer. Amplification (PCR) and sequencing were carried out using the methods described in Pridgeon et al. [7]. The chromatograms were assembled and edited with Sequencher v. 4.8 (Gene Codes Corp., Ann Arbor, Michigan, USA).

### Sequence alignment and cladistic analysis

Alignment of the sequences was carried out using the default settings of the online version of the program MAFFT v. 6 [10], with minor subsequent manual adjustment. Individual gap positions were treated as missing data and all characters were treated as unordered with equal weights. Pridgeon et al. [7] found no evidence of conflict among the trees resulting from separate analyses of the *matK/trnK*, *trnL/trnF* and ITS regions. Accordingly, we analyzed all three data sets in combination, conducting a parsimony analysis using the program PAUP* version 4.02b [11]. The analysis consisted of a heuristic search with 1000 replicates of random taxon addition for the starting trees and tree rearrangements using tree bisection-reconnection (“TBR”) branch swapping, saving all most-parsimonious trees (MPTs). Clade support was assessed by means of a nonparametric bootstrap analysis [12], consisting of 1000 bootstrap replicates, each with 20 heuristic replicates with random taxon addition and TBR branch swapping, saving up to 20 MPTs from each replicate.

## Results and Discussion

### Taxonomic treatment

#### Stelis zootrophionoides

**Figure 1:**
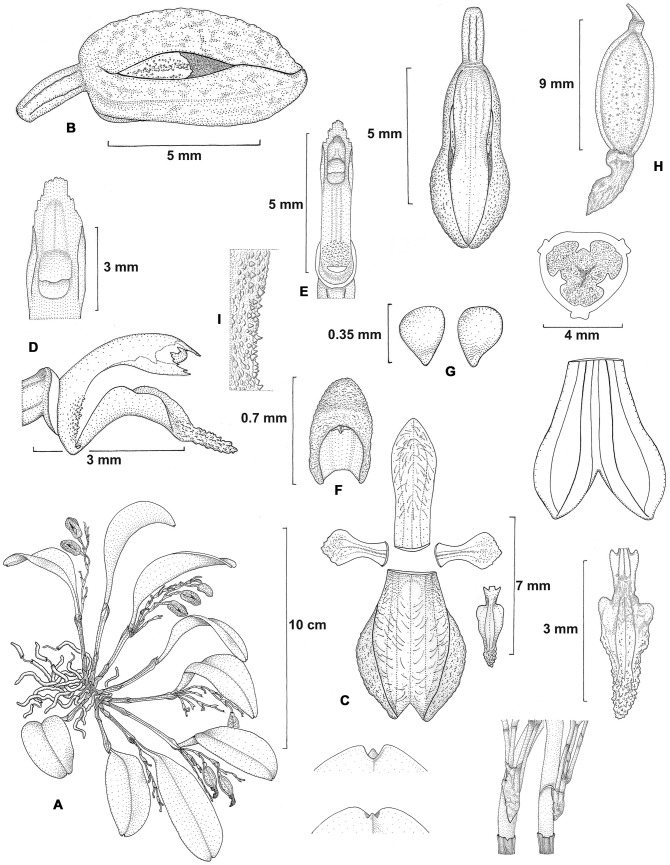
*Stelis zootrophionoides*. A) habit, B) flower, C) dissected flower, D) column and lip lateral view, E) column ventral view, F) anther cap. G, pollinia, H) fruit, I) detail of the column base. Drawn by SE Ramos-Castro from *Castañeda-Zárate & SE Ramos-Castro 448*.

**Figure 2:**
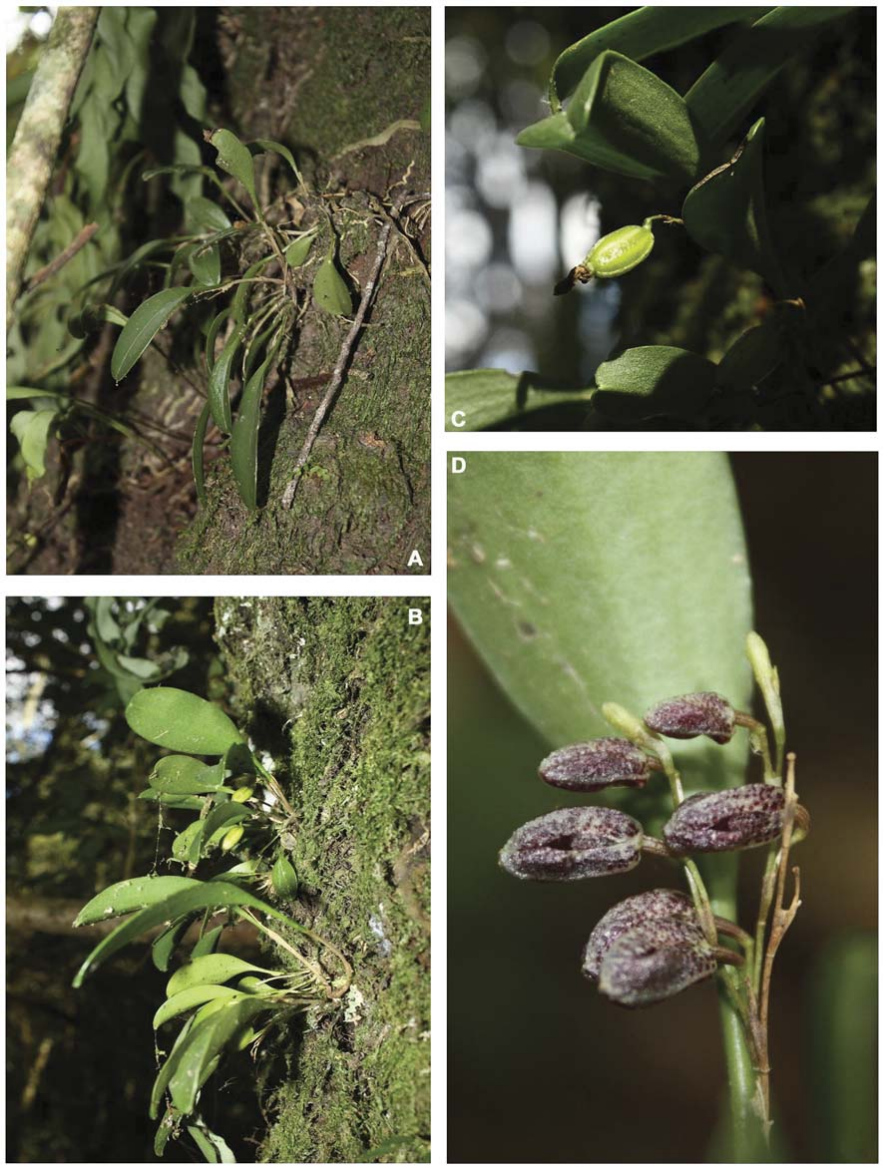
*Stelis zootrophionoides*. A, B) plants *in situ* during the season of vegetative growth, C) fruit, D) inflorescence. (Photographs A,D by SE Ramos-Castro, and B,C by M Castañeda-Zárate).

Castañeda-Zárate & Ramos-Castro, sp. nov. (Figs. 1, 2)

Type: MEXICO. Chiapas: Municipio San Cristóbal de Las Casas, Reserva Ecológica Huitepec, cloud forest, epiphytic on oak, rare, 5 September 2010, *M. Castañeda-Zárate & S.E. Ramos-Castro 448* (HOLOTYPE: MEXU; ISOTYPES: CHIP, MO, OAX, XAL).

Epiphytic, caespitose, herb up to 13.5 cm in height. Roots thin, terete, flexuous, white, 0.7–1.2 mm in diameter. Rhizome short, covered by tubular, scarious sheaths. Stems straight, thin, formed by 2 internodes, 2–5.5 cm long, 0.9–1.5 cm in diameter, with a thickened ring about 2.5–5 mm below the apex; covered by tubular sheaths, these obtuse, dilated towards the apex, carinate, mucronate, scarious, brownish-red when dry, lowermost sheath 0.9–2 cm long, uppermost sheath 1.6–3 cm long. Leaves oblanceolate to elliptic, rounded and minutely trilobed with mucronate apex, carinate on the abaxial surface, 3.8–8.4 cm long, 1.3–2.1 cm wide; petiole short, canaliculate, 10–15 mm long; blade fleshy, slightly arched. Inflorescence arising from the ring of the stem, racemose, shorter than the leaf, 1–3 per flowering season, 1.2–7 cm long, covered at the base by a spathaceous bract, the latter triangular, conduplicate, carinate, 3–5 mm long; peduncle straight, cylindrical, 4–13 mm long, 0.4–0.6 mm in diameter, covered by 2 tubular sheaths, these membranaceous, imbricating, obtuse, dilated and oblique towards the apex, 1.4–2.2 mm long; rachis slightly flexuous, elongating progressively towards the apex as new flowers develop, with up to 12 flowers, 1–2 open at a time. Floral bracts obliquely infundibuliform, obtuse, shortly apiculate, carinate, membranaceous, 2.7–3.8 mm long. Ovary slightly arched, thickened towards the apex, trigonous, glabrous, purple, 2.5–3.5 mm long, 0.8–1 mm diameter, articulate with the pedicel; pedicel united to the rachis above the base of floral bract, straight, cylindrical, thickened towards the apex, longer than the floral bract, 3.7–7 mm long, 0.4–0.6 mm in diameter. Flowers 4–8.1 mm long, resupinate, slightly pendent, the apex of the dorsal sepal adhered to the apex of the synsepal, forming two lateral, window-like openings; floral segments and column creamy yellow or greenish yellow, densely blotched with dark purple, anther white. Dorsal sepal united to the lateral sepals near the base, incurved, conduplicate, oblong-lanceolate, obtuse, fleshy, 3-veined, keeled on the outer surface, 6.8–7.3 mm long, 2.2–2.6 mm wide, sparsely pilose on the inner surface above the middle, hairs purple. Lateral sepals fused by two-thirds of their length to form a cymbiform, bifid synsepal, 6.5–7.1 mm long, 5.4–5.7 mm wide; the base of the synsepal forms a chin in which fits the column foot; lateral sepal apices obtuse, shortly apiculate and slightly oblique, 3-veined, keeled on the outer surface, sparsely pilose on the inner surface, hairs purple. Petals nearly parallel to the column, obovate-spatulate, fleshy, 3-veined, carinate at the abaxial surface, with rounded lateral angles, apical margins slightly erose, 2.5–3.6 mm long, 1.5–1.8 mm wide. Lip recurved, conduplicate, 3-veined, unguiculate, acute, margins erect on the proximal two-thirds, fleshy, 3.3–3.6 mm long, 0.7–1 mm wide; claw oblong, provided at each side of the base with a retrorse auricle, 1 mm long, 0.7 mm wide; blade sagittate when spread out, slightly crenulate at the margins, inner surface papillose; sides obtuse, provided with a submarginal callus, slightly thickened. Column slender, arcuate, winged, channeled ventrally, 2.8–3.2 mm long; wings erect, 0.6–0.7 mm wide; clinandrium surpassing the anther, dentate; column foot elongate, papillose at the apex. Stigma ventral, subquadrate, concave, covered by a viscous substance. Rostellum laminar, convex. Anther ventral, oblong-ovoid, two-celled, papillose-verrucose, 0.7 mm long, 0.5 mm wide. Pollinarium formed by 2 obovoid, laterally compressed, yellowish pollinia, 0.35–0.4 mm long, provided with granulose caudicles. Capsule ellipsoid, trigonous, with persistent perianth, 8.2–10 mm long, 3.7–4.9 mm in diameter.

#### Distribution and habitat

*Stelis zootrophionoides* is known from a single location in the central highlands of Chiapas, Mexico. The only population recorded was found growing epiphytically in humus accumulations on trunks of *Quercus laurina* in a cloud forest at 2,554 m elevation. Other epiphytes sharing the same habitat are *Peperomia quadrifolia* (L.) Kunth, *Epidendrum propinquum* A.Rich. & Galeotti, and various bryophytes and ferns such as *Campyloneurum angustifolium* (Sw.) Fée, *Dryopteris parallelogramma* (Kunze) Alston, and *Pleopeltis macrocarpa* (Bory ex Willd.) Kaulf. var. *interjecta* (Weath) A.R.Sm.

#### Phenology

Flowering occurs from July to September; developing fruits were observed in August, and seed dispersal occurred between October and December.

#### Conservation status

So far, this species is known from only one population and we consider it as rare, although additional field studies are required to ascertain its conservation status more objectively. It was located within a private protected area, the Reserva Ecológica Cerro Huitepec; however, outside the reserve natural vegetation is being strongly affected by logging and collecting of epiphytes [13]. It is interesting to note that the new species was discovered in a relatively accessible area and it was immediately obvious that it represented a novelty for the Mexican orchid flora, which highlights the fact that there are still species waiting to be discovered, while the remnant habitats are being lost rapidly.

#### Etymology

The specific epithet refers to the resemblance of the flowers to those of the genus *Zootrophion*; the latter was derived from Greek *zootrophion*, “a menagerie”, in allusion to the flowers that resemble the heads of animals [14].

#### Species recognition

*Stelis zootrophionoides* is unique among Mexican species of this genus by its raceme shorter than the leaf, with successive flowers whose sepals united at their apices leave lateral openings. Vegetatively, *S. zootrophionoides* is similar to *Stelis nigriflora*, *S. retusa* (Lex.) Pridgeon & M.W.Chase, and *S. sotoarenasii* Solano. *Stelis nigriflora* is distinguished from *S. zootrophionoides* by its larger plants, leaves spotted with purple on the abaxial surface, and abbreviated raceme; moreover, it has a restricted distribution around Tepoztlán, Morelos [15]. *Stelis retusa* and *S. sotoarenasii* are similar to one another and both differ from *S. zootrophionoides* in their narrower leaves with very short petiole and free sepal apices; *S. retusa* is found in the Transverse Volcanic Belt and the Sierra Madre del Sur [16], whereas *S. sotoarenasii* is known only from the mountains surrounding the central valleys of Oaxaca [5]. The only other Mexican or Central American species of *Stelis* in which the sepals adhere to one another at their apices is *Stelis jalapensis* (Kraenzl) Pridgeon & M.W.Chase, but this is a more robust plant with a many-flowered inflorescence much longer than the leaf, larger, pendulous flowers with sepals ≥10 mm long, sepals prominently ciliate and adhered only at their apex, and lateral sepals completely connate forming an entire, acute synsepal [15], [17].

**Figure 3:**
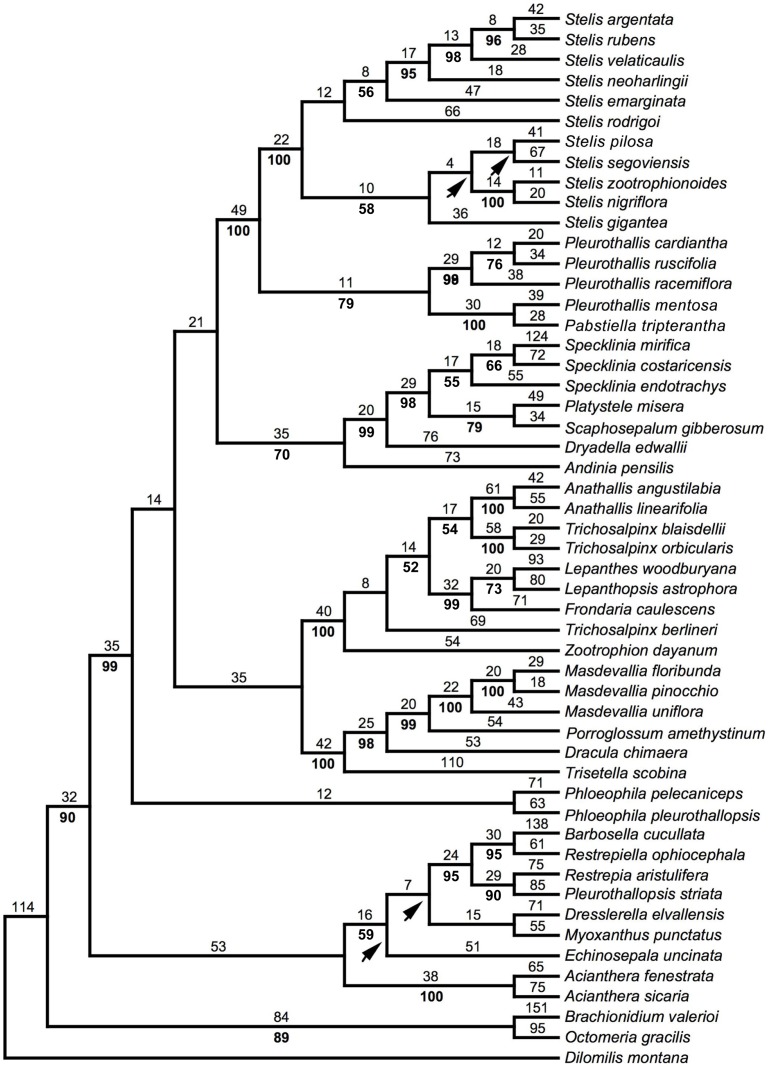
Phylogenetic tree. One of the four most parsimonious trees found by the combined analysis of *matK/trnK*, *trnL/trnF*, and nrITS DNA sequences. Numbers above branches are number of changes, numbers below branches are bootstrap percentages. The arrowheads point to clades that collapse in the strict consensus.

In our phylogenetic analysis, the aligned matrix consisted of 4392 characters, of which 1683 (38%) were variable and 868 (20%) were parsimony-informative. The analysis found four MPTs with a length of 4258 steps, consistency index (excluding uninformative characters) of 0.41, and retention index of 0.52. One of the four MPTs, on which bootstrap percentages are indicated, is shown in Figure 3. Overall, relationships agree with those recovered in the phylogenetic analyses of Pridgeon et al. [7]. *Stelis zootrophionoides* is embedded in strongly supported *Stelis* sensu Pridgeon and Chase [18], being strongly supported as sister to *S. nigriflora*. This species-pair is in turn sister to *S. pilosa* Pridgeon & M.W.Chase/*S. segoviensis* (Rchb.f.) Pridgeon & M.W.Chase, although this group collapsed in the strict consensus. This whole clade was recovered as sister to a weakly supported group including successively *S. rodrigoi* (Luer) Pridgeon & M.W.Chase, *S. emarginata* (Lindl.) Soto Arenas & Solano, *S. neoharlingii* (Garay) Pridgeon & M.W.Chase, *S. velaticaulis* (Rchb.f.) Pridgeon & M.W.Chase, and *S. rubens*/*S.argentata* Lindl.

The most salient feature distinguishing *S. zootrophionoides* from its close relatives is the adhesion of the distal portions of the sepals. As mentioned earlier, adhesion of the sepal apices is found in other distantly related groups of Pleurothallidinae [19]. Such similarity might be the result of use of the same or similar pollinators, as several studies have shown that homoplasy in floral attributes involved in pollination among unrelated clades is a widespread phenomenon in the Orchidaceae [20], [21]. According to the pollination syndromes concept, flowers can converge on a suite of floral traits (i.e. flower size, color, odor, rewards) associated with the attraction and utilization of a specific group of animals as pollinators [22]. Pleurothallidinae is mainly a group pollinated by small flies (myophily) of families Drosophilidae, Chlorophidae and Phoridae [23], [24], and the myophillous syndrome is characterized generally by bowl-shaped or flat flowers with windows or filiform appendages [25].

## Supporting Information

Table S1 **Taxa analyzed, voucher information or literature reference, and GenBank accessions for the DNA sequences.**
(DOCX)
