# Supplementary material for: Stelis zootrophionoides (Orchidaceae: Pleurothallidinae), a New Species from Mexico
Source: PLoS One. 2012 Nov 7;7(11):e48822. doi: 10.1371/journal.pone.0048822 (PMC3492219; doi:10.1371/journal.pone.0048822)
Supplement: Table S1 — Taxa analyzed, voucher information or literature reference, and GenBank accessions for the DNA sequences. (DOCX) [file pone.0048822.s001.docx]

**Table S1**. Taxa analyzed, voucher information or literature reference, and GenBank accessions for the DNA sequences.

|  |  | GenBank accessions | | |
| --- | --- | --- | --- | --- |
| Taxon | Voucher or reference | *matK/trnK* | *trnL‒trnF* | ITS |
| *Acianthera fenestrata* (Barb.Rodr.) Pridgeon & M.W.Chase | Pridgeon et al. (2001) *J & L Orchids s.n*., unvouchered | AF265468 | AF265518 | AF262857 |
| *Acianthera sicaria* (Lindl.) Pridgeon & M.W.Chase | Pridgeon et al. (2001) *Manning 950742* (K), Kew Spirit 60903 | AF302648 | AF276026 | AF262848 |
| *Anathallis angustilabia* (Schltr.) Pridgeon & M.W.Chase | Pridgeon et al. (2001) *Manning 890604* (K), Kew Spirit 60905 | AF302647 | AF293438 | AF262868 |
| *Anathallis linearifolia* (Cogn). Pridgeon & M.W.Chase | Pridgeon et al. (2001) *Hermans 2336* (K), Kew Spirit 569751 | AF265473 | AF265495 | AF262869 |
| *Andinia pensilis* (Schltr.) Luer | Pridgeon et al. (2001) *Hermans 4323* (K) | AF265455 | AF265502 | AF262826 |
| *Pleurothallis mentosa* Barb. Rodr. | Pridgeon et al. (2001) *J & L Orchids s.n*., unvouchered | AF265486 | AF293435 | AF262864 |
| *Pabstiella tripterantha* (Rchb.f.) F. Barros | Pridgeon et al. (2001) *Scardefield s.n.* Kew Spirit 62103 | AF302649 | AF293439 | AF275694 |
| *Barbosella cucullata* (Lindl.) Schltr. | Pridgeon et al. (2001) *Kew 1997‒5285* (K) | AF265483 | AF265525 | AF262815 |
| *Brachionidium valerioi* Ames & C.Schweinf. | Pridgeon et al. (2001) *J & L Orchids s.n*., unvouchered, *Manning 980403* (K) | AF265488 | AF291102 | AF262913 |
| *Dilomilis montana* Summerh. | Pridgeon et al. (2001) *Chase s.n*., unvouchered | AF263765 | AF266967 | AF262915 |
| *Dracula chimaera* (Rchb.f.) Luer | Pridgeon et al. (2001) *Hermans 1357* (K), Kew Spirit 58741 | AF265444 | AF265489 | AF262766 |
| *Dresslerella elvallensis* Luer | Pridgeon et al. (2001) *J & L Orchids s.n*., Kew Spirit 60155 | AF265477 | AF265521 | AF262901 |
| *Dryadella edwallii* (Cogn.) Luer | Pridgeon et al. (2001) *Chase s.n*., unvouchered | AF265454 | AF265505 | AF262824 |
| *Echinosepala uncinata* (Fawc.) Pridgeon & M.W.Chae | Pridgeon et al. (2001) *Kew 1980‒3412* (K) | AF265478 | AF265520 | AF262904 |
| *Frondaria caulescens* (Lindl.) Luer | Pridgeon et al. (2001) *Luer 18778* (K), Kew Spirit 61336 | AF265471 | AF265528 | AF262914 |
| *Lepanthes woodburyana* Stimson | Pridgeon et al. (2001) *Hermans 2931* (K), Kew Spirit 57773 | AF265472 | AF265494 | AF262890 |
| *Lepanthopsis astrophora* Garay | Pridgeon et al. (2001) *Manning 921101* (K), Kew Spirit 61340 | AF265487 | AF265493 | AF262893 |
| *Masdevallia floribunda* Lindl. | Pridgeon et al. (2001) *Chase s.n*., unvouchered | AY368416 | AF266966 | AF260146 |
| *Masdevallia pinocchio* Luer & Andreetta | Pridgeon et al. (2001) *Hermans 2379*(K), Kew Spirit 56945 | AF265445 | AF293433 | AF262778 |
| *Masdevallia uniflora* Ruiz & Pav. | Pridgeon et al. (2001) *Kew 1997‒5356* (K) | AF265446 | AF265490 | AF262769 |
| *Myoxanthus punctatus* (Barb.Rodr.) Luer | Pridgeon et al. (2001) *Kew 1970‒3331* (K), Kew Spirit 60911 | AF265479 | AF265519 | AF262885 |
| *Octomeria gracilis* G.Lodd. ex Lindl. | Pridgeon et al. (2001) *Hermans 2334* (K), Kew Spirit 58256 | AF265484 | AF265526 | AF262911 |
| *Phloeophila pelecaniceps* (Luer) Pridgeon & M.W.Chase | Pridgeon et al. (2001) *Hermans 3662* (K) | AF265450 | AF265512 | AF262810 |
| *Phloeophila pleurothallopsis* (Kraenzl.) Pridgeon & M.W.Chase | Pridgeon et al. (2001) *Hermans 2140* (K), *Manning 961086* (K), Kew Spirit 60908 | AF265451 | AF265511 | AF262811 |
| *Platystele misera* (Lindl.) Garay | Pridgeon et al. (2001) *Manning 890811* (K), Kew Spirit 61338 | AF265470 | AF265504 | AF262823 |
| *Pleurothallis cardiantha* Rchb.f. | Pridgeon et al. (2001) *Hermans 2950* (K) | AF265462 | AF265501 | AF262832 |
| *Pleurothallis racemiflora* (Sw.) Lindl. ex Hook. | ^a^Higgins et al. (2003) *FLAS 198267* | AY396076 | AY422379 | AY008477 |
| *Pleurothallis ruscifolia* (Jacq.) R.Br. | Pridgeon et al. (2001) *Hermans 2625* (K), Kew Spirit 60833 | AF265463 | AF265500 | AF262836 |
| *Pleurothallopsis striata* (Luer & R.Escobar) Prodgeon & M.W.Chase | Pridgeon et al. (2001) *Hermans 2652* (K), Kew Spirit 56922 | AF265480 | AF265524 | AF262910 |
| *Porroglossum amethystinum* (Rchb.f.) Garay | Pridgeon et al. (2001) *Kew 1997‒5400* (K) | AF265448 | AF265491 | AF262804 |
| *Restrepia aristulifera* Garay & Dunst. | Pridgeon et al. (2001) *Hermans 2639* (K), Kew Spirit 58144 | AF265481 | AF265522 | AF262907 |
| *Restrepiella ophiocephala* (Lindl.) Garay & Dunst. | Pridgeon et al. (2001) *Chase s.n*., unvouchered | AF265482 | AY422378 | AF262909 |
| *Scaphosepalum gibberosum* (Rchb.f.) Rolfe | Pridgeon et al. (2001) *Hermans 2366* (K), Kew Spirit 57889 | AF265458 | AF265503 | AF262817 |
| *Specklinia costaricensis* (Rolfe) Pridgeon & M.W.Chase | Pridgeon et al. (2001) *Kew 1997‒7405*, Kew Spirit 60907 | AF265459 | AF265506 | AF262862 |
| *Specklinia endotrachys* (Rchb.f.) Pridgeon & M.W.Chase | Pridgeon et al. (2001) *Hermans 2840* (K) | AF265456 | AF265508 | AF262859 |
| *Specklinia mirifica* Pridgeon & M.W.Chase | Pridgeon et al. (2001) *J & L Orchids s.n*., unvouchered | AF265457 | AF265507 | AF262865 |
| *Stelis argentata* Lindl. | Pridgeon et al. (2001) *Kew 1984‒4053*, Kew Spirit 60886 | AF265464 | AF265510 | AF262878 |
| *Stelis emarginata* (Lindl.) Soto Arenas & Solano | Pridgeon et al. (2001) *Kew 1973‒3662*, (K) Kew Spirit 60239 | AF265466 | AF265514 | AF262845 |
| *Stelis gigantea* Pridgeon & M.W.Chase | Pridgeon et al. (2001) *J & L Orchids s.n*., unvouchered | AF265461 | AF265513 | AF262843 |
| *Stelis neoharlingii* (Garay) Pridgeon & M.W.Chase | Pridgeon et al. (2001) *Kew 1957‒46701*, Kew Spirit 60883 | AF265465 | AF265509 | AF262846 |
| *Stelis nigriflora* (L.O.Williams) Pridgeon & M.W.Chase | ^b^México, Morelos *Reyes 5684* (MEXU) | FR837538 | FR837541 | FR837535 |
| *Stelis pilosa* Pridgeon & M.W.Chase | Pridgeon et al. (2001) *Hermans 2039* (K), Kew Spirit 56872 | AF265467 | AF265517 | AF262831 |
| *Stelis rodrigoi* (Luer) Pridgeon & M.W.Chase | Pridgeon et al. (2001) *Hermans 1926* (K), Kew Spirit 57031 | AF265460 | AF265516 | AF262829 |
| *Stelis rubens* Schltr. | ^b^Mexico, Puebla *Salazar et al. 6554bis* (MEXU) | FR837536 | FR837539 | FR837533 |
| *Stelis segoviensis* (Rchb.f.) Pridgeon & M.W.Chase | Pridgeon et al. (2001) *Manning 890812* (K), Kew Spirit 60915 | AF276313 | AF265515 | AF262866 |
| *Stelis velaticaulis* (Rchb.f.) Pridgeon & M.W.Chase | Pridgeon et al. (2001) *Kew 1995‒40102* (K) | AF302646 | AF293437 | AF262847 |
| *Stelis zootrophionoides* Castañeda‒Zárate & Ramos‒Castro | ^b^México, Chiapas *Castañeda‒Zárate & S. Ramos‒Castro 448* (MEXU) | FR837537 | FR837540 | FR837534 |
| *Trichosalpinx berlineri* (Luer) Luer | Pridgeon et al. (2001) *Hermans 1605* (K), Kew Spirit 56862 | AF265475 | AF265497 | AF262900 |
| *Trichosalpinx blaisdellii* (S.Wats.) Luer | Pridgeon et al. (2001) *Kew 1997‒7412* (K) | AF265474 | AF265498 | AF262887 |
| *Trichosalpinx orbicularis* (Lindl.) Luer | Pridgeon et al. (2001) *Hermans 1349* (K), Kew Spirit 56883 | AF265476 | AF265496 | AF262886 |
| *Trisetella scobina* Luer | Pridgeon et al. (2001) *Kew 1997‒5449* (K), Kew Spirit 60914 | AF265449 | AF265492 | AF262808 |
| *Zootrophion dayanum* (Rchb.f.) Luer | Pridgeon et al. (2001) *Hermans2142* (K), Kew Spirit 60145 | AF265452 | AF265499 | AF262895 |

^a^ Reference: Higgins WE, van den Berg C, Whitten WM (2003) A combined molecular phylogeny of Encyclia (Orchidaceae) and relationships within Laeliinae. Selbyana 24: 165‒179.

^b^ Sequences produced in this work.
